# Use of linear mixed models for genetic evaluation of gestation length and birth weight allowing for heavy-tailed residual effects

**DOI:** 10.1186/1297-9686-42-26

**Published:** 2010-06-30

**Authors:** Kadir Kizilkaya, Dorian J Garrick, Rohan L Fernando, Burcu Mestav, Mehmet A Yildiz

**Affiliations:** 1 Department of Animal Science, Iowa State University, Ames, IA 50011 USA; 2 Department of Animal Science, Adnan Menderes University, Aydin 09100 Turkey; 3 Institute of Veterinary, Animal and Biomedical Sciences, Massey University, Palmerston North, New Zealand; 4 Department of Animal Science, University of Ankara, Diskapi Ankara 06110 Turkey

## Abstract

**Background:**

The distribution of residual effects in linear mixed models in animal breeding applications is typically assumed normal, which makes inferences vulnerable to outlier observations. In order to mute the impact of outliers, one option is to fit models with residuals having a heavy-tailed distribution. Here, a Student's-*t *model was considered for the distribution of the residuals with the degrees of freedom treated as unknown. Bayesian inference was used to investigate a bivariate Student's-*t *(BS*t*) model using Markov chain Monte Carlo methods in a simulation study and analysing field data for gestation length and birth weight permitted to study the practical implications of fitting heavy-tailed distributions for residuals in linear mixed models.

**Methods:**

In the simulation study, bivariate residuals were generated using Student's-*t *distribution with 4 or 12 degrees of freedom, or a normal distribution. Sire models with bivariate Student's-*t *or normal residuals were fitted to each simulated dataset using a hierarchical Bayesian approach. For the field data, consisting of gestation length and birth weight records on 7,883 Italian Piemontese cattle, a sire-maternal grandsire model including fixed effects of sex-age of dam and uncorrelated random herd-year-season effects were fitted using a hierarchical Bayesian approach. Residuals were defined to follow bivariate normal or Student's-*t *distributions with unknown degrees of freedom.

**Results:**

Posterior mean estimates of degrees of freedom parameters seemed to be accurate and unbiased in the simulation study. Estimates of sire and herd variances were similar, if not identical, across fitted models. In the field data, there was strong support based on predictive log-likelihood values for the Student's-*t *error model. Most of the posterior density for degrees of freedom was below 4. Posterior means of direct and maternal heritabilities for birth weight were smaller in the Student's-*t *model than those in the normal model. Re-rankings of sires were observed between heavy-tailed and normal models.

**Conclusions:**

Reliable estimates of degrees of freedom were obtained in all simulated heavy-tailed and normal datasets. The predictive log-likelihood was able to distinguish the correct model among the models fitted to heavy-tailed datasets. There was no disadvantage of fitting a heavy-tailed model when the true model was normal. Predictive log-likelihood values indicated that heavy-tailed models with low degrees of freedom values fitted gestation length and birth weight data better than a model with normally distributed residuals.

Heavy-tailed and normal models resulted in different estimates of direct and maternal heritabilities, and different sire rankings. Heavy-tailed models may be more appropriate for reliable estimation of genetic parameters from field data.

## Background

Animal breeding applications commonly involve the fitting of linear mixed models in order to estimate genetic and phenotypic variation or to predict the genetic merit of selection candidates. Measurement errors and other sources of random non-genetic variation comprise the residual term, the effects of which are often assumed to be normally distributed with zero mean and common variance. These assumptions may make inferences vulnerable to the presence of outliers [1,2]. Heavy-tailed densities (such as Student's-*t *distribution) are viable alternatives to the normal distribution, and provide robustness against unusual or outlying observations when used to model the densities of residual effects. In the event that the degrees of freedom are estimated to be large, i.e. in excess of 30, these methods converge to normally distributed residuals [3].

Mixed effects linear models with Student's-*t *distributed error effects have been applied to mute the impact of residual outliers, for example in a situation where preferential treatment of some individuals was suspected [4]. Von Rohr and Hoeschele [5] have demonstrated the application of a Student's-*t *sampling model under four different error distributions in statistical mapping of quantitative trait loci (QTL). They have determined that additive and dominance QTL and residual variance estimates are much closer to the simulated true values when the data itself is heavy-tailed and the analysis is performed with the skewed Student's-*t *model rather than with a normal model. Rosa et al. [6] have analyzed birth weight in a reproductive toxicology study and compared normal as well as robust mixed linear models based on Student's-*t *distribution, Slash or contaminated normal error distributions. Marginal posterior densities of degrees of freedom for the Student's-*t *and Slash error distributions are concentrated about single digit values, suggesting the inadequacy of the normal distribution for modelling residual effects. The heavy-tailed distributions result in significantly better fit than a normal distribution. Kizilkaya et al. [3] have applied threshold models with normal or Student's-*t *link functions for the genetic analysis of calving ease scores and they have shown that predictive log-likelihoods strongly favour a Student's-*t *model with low degrees of freedom in comparison with a normal distribution. Cardoso et al. [7] have used heavy-tailed distributions to study residual heteroskedasticity in beef cattle and have found that a Student's-*t *model significantly improves predictive log-likelihood value. Chang et al. [8] have compared multivariate heavy-tailed and probit threshold models in the analysis of clinical mastitis in first lactation cows, and have shown that a model comparison strongly supports the multivariate Slash and Student's-*t *models with low degrees of freedom over the probit model. The objectives of this research were to 1) examine by simulation if Bayesian inference under a bivariate Student's-*t *distribution of residuals can accommodate models with either light-tailed or heavy-tailed residuals, and 2) investigate the practical implications of fitting a Student's-*t *distribution with unknown degrees of freedom for the residuals in bivariate field data. In both cases, results were compared to those from the conventional approach of assuming bivariate normal (BN) residuals.

## Methods

We first present the theory and methods for multiple traits that are applicable to both the simulation and the analysis of field data on gestation length and birth weight using a model that accommodates heavy-tailed residuals.

### Statistical model

A linear mixed model for animal *i *is(1)

where **y**_*i *_= (*y*_*i*,1 _... *y*_*i*, *m*_)' is a vector of phenotypic values of animal *i *for *m *traits, **b **is a vector of fixed effects, **a **is a vector of random genetic effects, **h **is a vector of uncorrelated random effects such as herd effects, **X**_*i*_, **Z**_*i *_and **W**_*i*_, are design matrices for animal *i*, corresponding to the vectors of the fixed effects (**b**), random genetic effects (**a**), and uncorrelated random effects (**h**).

Conventional analyses might assume the vector ϵ_*i *_in equation (1) is multivariate normally distributed (*N*(**0**, **R**_0_)), where

In contrast, we assume ϵ_*i *_in (1) is multivariate heavy- or light-tailed by expressing the residual in the usual manner but divided by a scalar random variable that varies for each animal *i *but is consistent across the traits. That is,(2)

where *λ*_*i *_in equation (2) is a positive random variable [9]. Values of *λ*_*i *_approaching 0 produce heavy-tailed residuals for both traits, whereas values exceeding 1 would produce light-tails. The marginal density of ϵ_*i *_is a multivariate Student's-*t *density with scale parameter **R**_**0 **_and df *ν*, such that the marginal residual variance becomes  [4,7,9].

### Prior and full conditional posterior distributions

A flat prior was assumed for the fixed effects (**b**). Genetic effects (**a**) were assumed to be distributed as multivariate normal, with null mean vector and (co)variance matrix **A **⊗ **G**_**0 **_where **A **is the numerator relationship matrix and ⊗ denotes the Kronecker product [10]. Uncorrelated random effects and residuals were assumed to follow multivariate normal distributions with null means and (co)variance matrices **I **⊗ **H**_**0 **_and **I **⊗ **R**_**0 **_where **I **is the identity matrix. Flat prior distributions were assigned to **G**_**0**_, **H**_**0 **_and **R**_**0**_.

The multivariate normal distribution requires no distributional specification of *λ*_*i *_in equation (2), because *λ*_*i *_= 1 for all *i *= 1, 2,...,*n*. The distribution of *λ*_*i *_in equation (2) for multivariate Student's-*t *is a *Gamma*(*ν*/2, *ν*/2) distribution with density function

Where *λ*_*i *_> 0, Γ(.) is the standard *Gamma *function, *i *= 1, 2,...,*n *and *ν *> 0. A prior of  for *ν *> 0 was assigned to *ν *[3].

Inferences on parameters of interest can be made from the posterior distributions constructed using MCMC methods such as Gibbs sampling or Metropolis-Hastings [11-13]. The fully conditional posterior distributions of each of the unknown parameters are used to generate proposal samples from the target distribution (the joint posterior). The fully conditional posterior distributions of fixed (**b**), genetic (**a**) and uncorrelated random (**h**) effects are multivariate normal with mean  and covariance matrix **C**, where  are solutions to Henderson's mixed model equations constructed with heterogeneous residual variances,  and **C **is the inverse of this mixed-model coefficient matrix [4]. The (co)variance matrices **G**_**0**_, **H**_**0 **_and **R**_**0 **_have inverse Wishart conditional posterior distributions, which can also be constructed from  where  is solution for *λ*_*i *_[9].

The fully conditional posterior distributions of *λ*_*i *_for the multivariate Student's-*t *model is

where **e **= **y**_*i *_- **X**_*i*_**b **- **Z**_*i*_**a **- **W**_*i*_**h**.

The fully conditional posterior distribution of df *ν *for the multivariate Student's-*t *model does not have a standard form, and so a sampling strategy for nonstandard distributions is required. A random-walk Metropolis-Hastings (MH) algorithm was used to draw samples for *ν *[11]. In the MH algorithm, a normal density with expectation equal to the parameter value from the previous MCMC cycle was used as the proposal density. The MH acceptance ratio was tuned to intermediate rates (40-50%) during the MCMC burn-in period to optimize MCMC mixing [3]. Sampled values of *ν *< 2 were truncated to 2 so that covariance matrix, , for the residuals of (1) is defined.

#### Simulation study

A simulation study was carried out to validate Bayesian inference on the bivariate Student's-*t *models, and assess the ability of model choice criterion (predictive log-likelihood) to correctly choose the model with better fit. For this purpose, the simulation study was undertaken using three sire models to simulate the bivariate data, these models varying in the nature of the simulated residual effects. We refer to the model used to simulate the data as the true model. These three models were the bivariate normal which effectively has infinite *ν *(BN-∞) and the bivariate Student's-*t *model with *ν *= 4 or 12 (BS*t*-4, BS*t*-12). Ten replicated data sets were generated for each of the three true models. Phenotypes of 50 progeny from each of 50 unrelated sires for two traits, **y**_*i *_= (*y*_*i*, 1 _*y*_*i*, 2_)' were simulated using equation (1). The vector of fixed effects **b **only included a gender effect with *b*_1 _= (11 90)' for trait 1 and *b*_2 _= (38 32)' for trait 2. The random genetic effects (**a**) and uncorrelated random effects (**h**) included 50 sires and 100 herds, respectively, assuming:

where **G**_0 _is the sire (co)variance matrix,

and **H**_0 _is the herd (co)variance matrix

Residuals were assumed **e**_*i *_~ *N *(**0**, **R**_0_), where

Heritabilities of simulated traits were  and , respectively. For each animal *i*, *λ*_*i *_was 1 for BN-∞ or generated from *Gamma*(*ν*/2, *ν*/2) for BS*t *- *ν *with *ν *= 4, 12. Offspring were assigned to herd and gender groups by random sampling from a uniform distribution.

#### Gestation length and birth weight data

Gestation length (GL) up until first calving and the resultant calf birth weight (BW) data were recorded on the national population of Italian Piemontese cattle from January 1989 to July 1998 by Associazione Nazionale Allevatori Bovini di Razza Piemontese (ANABORAPI), Strada Trinità 32a, 12061 Carrù, Italy. Only herds represented by at least 100 records over that period were considered in the study [14], providing a total of 7,883 animals from 677 sires and 747 MGS. Table 1 summarizes the statistics for GL and BW. BS*t *and BN models given in equation (1) were used to analyze GL and BW data. The fixed effects (**b**) of dam age in months, sex of the calf, and their interaction were considered by combining eight different first-calf age group classes (20 to 23, 23 to 25, 25 to 27, 27 to 29, 29 to 31, 31 to 33, 33 to 35, and 35 to 38 months) with sex of calf for a total of 16 nominal age-sex subclasses. A total of 1,186 herd-year-season (HYS) subclasses were created from combinations of herd, year, and two different seasons (from November to April and from May to October) as in Carnier et al. [15] and Kizilkaya et al., [3] and treated as uncorrelated random effects (**h**) [14]. The range for number of observations in HYS subclasses was between 1 and 33, and average number of records for HYS effect was 7. The random genetic effects (**a**) included 1,929 sires (**s**) and MGS (**m**) from the pedigree file. While the number of observations ranged from 1 to 406, average observations for each sire in data file was 12. We also assumed:

**Table 1:** Summary statistics for gestation length (GL) and birth weight (BW) in Italian Piemontese cattle.

Trait	N	Mean	Minimum	Maximum	SD
GL (day)	7,883	290	260	320	8.1
BW (kg)	7,883	39.6	22	56	4.1

where **G**_0 _is the sire-MGS (co)variance matrix,

and **H**_0 _is the HYS (co)variance matrix,

### Marginal residual variances, heritabilities and genetic correlations

Residual scale parameters (**R**_0_) in heavy-tailed models cannot be directly compared with the residual (co)variance (**R**_0_) in the normal model, nor used in estimation of heritabilities, residual or phenotypic correlations. The scale parameters must be appropriately transformed into marginal residual (co)variance parameters  for BN and BS*t *models, using **R**_*E *_= **R**_0 _and  where *ν *> 2, respectively, given by Stranden and Gianola [4] and Cardoso et al. [7].

Heritabilities and genetic correlations are of interest from the perspective of direct and maternal effects in an animal model, but the fitted models for GL and BW included genetic effects for sire and MGS, and some fractions of the genetic effects were included in the residual terms. Transformations were applied to convert the sire-MGS parameters and estimates to their animal model equivalent. The additive genetic (co)variance matrix including direct (*D*) and maternal (*M*) genetic variances from sire-MGS model was obtained as **G**_*DM *_= **PG**_0_**P**' [16] where **G**_*DM *_is an additive genetic (co)variance matrix,

and **P **is an appropriate transformation matrix,

Direct and maternal () heritability, and genetic correlation () estimates were obtained from estimates of variance and covariance components according to:

and

Where *G *and *G' *= *D *or *M*, *i *and *k *for the trait of GL or BW and *j *for the model of BN or BS*t*.

Kendall rank correlations between posterior means of sire genetic effects obtained from the BN and BS*t *models were used to compare the ordering of the genetic evaluations of the sires for GL and BW [17]. Comparisons were also made between rank orders of the top 100 selection candidates from 1,929 animals in the pedigree file for the BN model.

### Model comparison

Model comparisons in the simulation study, and for the analysis of field data, were carried out using predictive log-likelihoods (PLL) from BN and BS*t *models. The PLL over all observations (*n*) under Model *M*_*k *_(*k *= BN or BS*t*) was obtained as:(3)

where  is the harmonic mean of *p*^-1^(**y**_*i*_|***θ***^(*j*)^, *M*_*k*_) across *G *MCMC samples [18]. A PLL difference exceeding 2.5 was used as indication of an important difference in model fit, following Raftery [19].

In the simulation study, the impact of alternative models was quantified by computing the correlations (r_**â, a**_) between the simulated true (**a**) and predicted (**â**) sire effects in each of the three fitted models. Further, the prediction error variance (PEV) V(**a **- **â**)) of the sire effects was calculated to provide an informative comparative assessment of model prediction performance. Higher correlations and lower prediction error variances will be associated with fitted models that are better at predicting breeding values than models with low correlations and high prediction error variance. Some fitted models might be significantly better than others from a likelihood framework, yet have little impact on selection response if they do not markedly change correlations. Minimizing the prediction error variances is important when investment decisions depend upon the magnitude of the sire predictions, not just the ranking of the sires.

### MCMC implementation

Graphical inspection (time series traces) of the chains along with Heidelberger and Welch Diagnostic [20] for the Gibbs output using CODA (Convergence Diagnostics and Output Analysis package in R) [21] were used to determine a common length of burn-in period. A burn-in period of 50,000 for simulated and field data analysis was defined as the number of cycles discarded at the start of the MCMC chain to ensure sampling from the correct marginal distributions. A further 50,000 post burn-in MCMC cycles in the simulated and field data analysis were generated for each of the BS*t *and BN models. Every successive post burn-in sample was retained, so that 50,000 samples were used to infer posterior distributions of unknown parameters. Posterior means of the parameters were obtained from their respective marginal posterior densities. Interval estimates were determined as posterior probability intervals (PPI) obtained from the 2.5 and 97.5 percentiles of each posterior density to provide 95% PPI. The effective number of independent samples (ESS) for each parameter was determined using the initial positive sequence estimator of Geyer [22] as adapted by Sorensen et al. [23].

## Results and discussion

### Simulation study

The predictive log-likelihood values in Table 2 were computed for BS*t *and BN models fitted to the simulated heavy-tailed and normal datasets. When the true model had residuals with heavy-tails, the fitted models with heavy-tails (BS*t*) were significantly better than the normal model (BN). When the true model had normally distributed residuals, all the fitted models performed equally well. The difference in PLL between the fitted models with heavy-tails and the normal model was inversely related to the degrees of freedom of the simulated residuals. Note that normally distributed residuals can be thought of as having infinite degrees of freedom, and in this case there were no differences between the fitted models.

**Table 2:** Comparisons of average predictive log-likelihood^1 ^(PLL) from ten replicates between bivariate Student's-*t *(BS*t*) and normal (BN) fitted models (in column) for different true simulated models (in rows) with varying residual degrees of freedom (DF).

	Fitted Model^3^	
	
True Model^2^-DF	BS*t*	BN
BS*t*-4	-1,483	-1,988
BS*t*-12	-718	-754
BN-∞	-284	-284

^1^Predictive log-likelihood values were reported after adding 14,000

^2^Used to simulate data

^3^Used in analysis of simulated data

Inference on *ν *based on BS*t *model analysis of BS*t*-4, BS*t*-12 and BN-∞ data sets is given in Table 3. Posterior means of *ν *seems sharp and unbiased, and the 95% posterior probability intervals for *ν *concentrated on low values for BS*t*-4 and BS*t*-12 data sets. Conversely, inference on *ν *for BN-∞ data was larger than 100, consistent with what was expected, and the 95% posterior probability interval was wider by concentrating on values higher than 30, indicating strong evidence of normally distributed data. Furthermore, relatively larger ESS of *ν *were obtained from BS*t*-4 and BS*t*-12 data sets when compared with that from BN-∞ data sets [3], indicating more samples would be needed to attain a minimum of 100 as advocated by Bink et al. [24] and Uimari et al. [25].

**Table 3:** Average posterior inference on degrees of freedom from ten replicates using the bivariate Student's-*t *(BS*t*) fitted model.

		BS*t *Fitted Model^2^		
		
True Parameters	True Model^1^	PM ± SE^3^	95% PPI^4^	ESS^5^
*ν *= 4	BS*t*-4	4.1 ± 0.06	[3.6, 4.6]	1,594
*ν *= 12	BS*t*-12	13.3 ± 1.18	[9.8, 19.1]	294
*ν *= ∞	BN-∞	2377 ± 654	[2140, 3365]	14

^1^Used to simulate data

^2^Used in analysis of simulated data

^3^Posterior mean ± Standard Error

^4^95% equal-tailed posterior probability interval based on the 2.5^*th *^and 97.5^*th *^percentiles of the posterior density

^5^Effective sample size

Tables 4, 5 and 6 summarize inferences on sire, herd and marginal error variances based on the replicated datasets from the three different populations, comparing BS*t *and BN fitted models. Large ESS were attained for sire, herd and marginal error variances, indicating stable MCMC inference. The 95% posterior probability intervals for sire and herd variance components from the three fitted models widely overlapped and included the true parameter values. Furthermore, the posterior means from the three fitted models were almost identical. When the true model was BS*t*-4 or BN-∞, inferences on marginal error (co)variance components using the BS*t *and BN fitted models were similar, found to be sharp and seemingly unbiased, and true parameter values were covered by 95% equal-tailed PPI of parameters (Table 6).

**Table 4:** Average posterior inference on sire (co)variances from ten replicates using the bivariate Student's-*t *(BS*t*) and normal (BN) fitted models with different residual degrees of freedom (DF).

		Fitted Model^2^					
		
		BS*t*			BN		
			
True Parameters	True Model^1^	PM ± SE^3^	95% PPI^4^	ESS^5^	PM ± SE	95% PPI	ESS
= 2.0	BS*t*-4	2.24 ± 0.16	[1.32, 3.62]	20,983	2.31 ± 0.18	[1.30, 3.83]	20,694
	BS*t*-12	2.44 ± 0.14	[1.48, 3.88]	24,767	2.39 ± 0.14	[1.44, 3.81]	25,794
	BN-∞	2.49 ± 0.17	[1.52, 3.93]	26,715	2.49 ± 0.17	[1.53, 3.93]	28,154
= 1.5	BS*t*-4	1.41 ± 0.17	[0.40, 2.76]	23,326	1.43 ± 0.18	[0.33, 2.89]	23,146
	BS*t*-12	1.93 ± 0.19	[0.83, 3.47]	27,428	1.89 ± 0.19	[0.80, 3.42]	28,683
	BN-∞	1.77 ± 0.22	[0.72, 3.23]	28,441	1.77 ± 0.21	[0.71, 3.22]	30,064
= 4.0	BS*t*-4	4.23 ± 0.30	[2.59, 6.70]	24,192	4.33 ± 0.33	[2.57, 6.99]	23,978
	BS*t*-12	4.77 ± 0.29	[2.97, 7.48]	27,674	4.80 ± 0.30	[2.98, 7.55]	29,102
	BN-∞	4.46 ± 0.39	[2.79, 6.96]	29,013	4.45 ± 0.39	[2.78, 6.98]	29,365

^1^Used to simulate data

^2^Used in analysis of simulated data

^3^Posterior mean ± Standard Error

^4^95% equal-tailed posterior probability interval based on the 2.5^*th *^and 97.5^*th *^percentiles of the posterior density

^5^Effective sample size

**Table 5:** Average posterior inference on herd variances from ten replicates using the bivariate Student's-*t *(BS*t*) and normal (BN) fitted models with different residual degrees of freedom (DF).

		Fitted Model^2^					
		
		BS*t*			BN		
			
True Parameters	True Model^1^	PM ± SE^3^	95% PPI^4^	ESS^5^	PM ± SE	95% PPI	ESS
= 1.5	BS*t*-4	1.71 ± 0.10	[1.07, 2.56]	12,027	1.74 ± 0.14	[1.01, 2.71]	10,437
	BS*t*-12	1.82 ± 0.12	[1.19, 2.65]	16,133	1.82 ± 0.12	[1.18, 2.65]	16,802
	BN-∞	1.71 ± 0.08	[1.12, 2.48]	17,543	1.70 ± 0.08	[1.12, 2.47]	17,537
= 6.0	BS*t*-4	6.33 ± 0.30	[4.47, 8.79]	24,120	6.49 ± 0.28	[4.47, 9.17]	22,810
	BS*t*-12	6.69 ± 0.28	[4.77, 9.22]	27,704	6.72 ± 0.24	[4.79, 9.28]	27,956
	BN-∞	6.33 ± 0.27	[4.55, 8.71]	29,800	6.33 ± 0.27	[4.55, 8.71]	29,881

^1^Used to simulate data

^2^Used in analysis of simulated data

^3^Posterior mean ± Standard Error

^4^95% equal-tailed posterior probability interval based on the 2.5^*th *^and 97.5^*th *^percentiles of the posterior density

^5^Effective sample size

**Table 6:** Average posterior inference on marginal error (co)variances from ten replicates using the bivariate Student's-*t *(BS*t*) and normal (BN) fitted models with different residual degrees of freedom (DF).

		Fitted Model^2^					
		
		BS*t*			BN		
			
True Parameters	True Model^1^	PM ± SE^3^	95% PPI^4^	ESS^5^	PM ± SE	95% PPI	ESS
= 30.0	BS*t*-4	30.45 ± 0.51	[27.44, 34.05]	3,336	30.29 ± 0.44	[28.61, 32.07]	42,430
= 18.0	BS*t*-12	17.87 ± 0.17	[16.75, 19.07]	9,516	17.82 ± 0.16	[16.83, 18.87]	43,135
= 15.0	BN-∞	14.94 ± 0.11	[14.10, 15.82]	43,387	14.94 ± 0.11	[14.11, 15.82]	43,204
= 8.0	BS*t*-4	8.20 ± 0.38	[6.45, 10.10]	11,384	8.37 ± 0.66	[6.95, 9.83]	44,553
= 4.8	BS*t*-12	4.61 ± 0.13	[3.69, 5.56]	32,416	4.57 ± 0.12	[3.72, 5.44]	44,370
= 4.0	BN-∞	3.94 ± 0.10	[3.23, 4.67]	43,729	3.94 ± 0.10	[3.23, 4.66]	44,238
= 40.0	BS*t*-4	40.07 ± 0.65	[35.98, 44.94]	3,566	39.57 ± 0.74	[37.37, 41.90]	45,168
= 24.0	BS*t*-12	24.60 ± 0.31	[23.04, 26.27]	10,145	24.54 ± 0.28	[23.17, 25.98]	45,130
= 20.0	BN-∞	20.13 ± 0.18	[19.00, 21.31]	42,782	20.12 ± 0.18	[19.00, 21.31]	45,079

^1^Used to simulate data

^2^Used in analysis of simulated data

^3^Posterior mean ± Standard Error

^4^95% equal-tailed posterior probability interval based on the 2.5^*th *^and 97.5^*th *^percentiles of the posterior density

^5^Effective sample size

Average correlations between true and estimated sire effects and average PEV from two replicates using BS*t *and BN fitted models are presented in Table 7 and 8. When the true model was BS*t*, both the correlation and PEV indicate that the heavy-tailed fitted models were superior, especially when the true value of *ν *= 4. When the true model was BN, all fitted models performed identically. In general, the accuracy and PEV results from BSt and BN models suggest that heavy-tailed fitted models can improve accuracy and PEV when the true model is heavy-tailed, but a robust Bayesian analysis using heavy-tailed models does not deteriorate accuracy and PEV if the true model is normal.

**Table 7:** Average correlations between true and predicted sire effects from ten replicates using the bivariate Student's-*t *(BS*t*) and normal (BN) fitted models with different residual degrees of freedom (DF).

	Fitted Model^2^			
	
	Trait1		Trait2	
		
True Model^1^-DF	BS*t*	BN	BS*t*	BN
BS*t*-4	0.90	0.87	0.92	0.90
BS*t*-12	0.93	0.93	0.95	0.95
BN-∞	0.94	0.94	0.95	0.95

^1^Used to simulate data

^2^Used in analysis of simulated data

**Table 8:** Prediction error variance of sire effects using the bivariate Student's-*t *(BS*t*) and normal (BN) fitted models with different residual degrees of freedom (DF).

	Fitted Model^2^			
	
	Trait1		Trait2	
		
True Model^1^-DF	BS*t*	BN	BS*t*	BN
BS*t*-4	0.36	0.44	0.51	0.67
BS*t*-12	0.29	0.30	0.41	0.44
BN-∞	0.23	0.23	0.33	0.33

^1^Used to simulate data

^2^Used in analysis of simulated data

### Application to gestation length and birth weight

#### Inference on degrees of freedom, variance components and heritabilities

The analyses produced PLL values for BS*t *and BN models of -47,006 and -48,006 respectively. The log-scale differences between model PLL values for BS*t *versus BN models were 1,000, which greatly exceeds 2.5 and decisively indicates the inadequacy of the normality assumption for the distribution of error terms. These results are in agreement with Chang et al. [8] and Cardoso et al. [17], who found that the Student's-*t *distribution was a better fit to the clinical mastitis data and postweaning gain data, respectively, compared to Slash and normal distributions.

The estimated ESS for *ν *is 1,227 and those for variance components are given in Tables 9, 10 and 11. The ESS for these parameters ranged from 323 to 15,789, indicating sufficient MCMC mixing. These values were found to be considerably higher than 100, which has been suggested as the minimum ESS for reliable statistical inference [24,25].

**Table 9:** Posterior inference on sire-MGS (co)variances for gestation length (GL) and birth weight (BW) using the bivariate Student's-*t *(BS*t*) and normal (BN) models.

	BS*t*			BN		
		
Parameters	PM^1^	95% PPI^2^	ESS^3^	PM	95% PPI	ESS
	8.42	[6.65, 10.43]	894	8.13	[6.27, 10.31]	384
	0.13	[-0.43, 0.72]	774	0.16	[-0.48, 0.81]	496
	2.75	[1.77, 3.76]	567	2.73	[1.63, 3.81]	323
	-0.54	[-1.04, -0.04]	524	-0.74	[-1.32, -0.21]	405
	1.02	[0.68, 1.43]	528	1.12	[0.75, 1.55]	550
	0.26	[-0.13, 0.69]	429	0.40	[-0.09, 0.90]	230
	0.36	[0.15, 0.58]	428	0.39	[0.18, 0.62]	484
	2.24	[1.47, 3.16]	389	2.04	[1.17, 3.05]	232
	0.27	[-0.03, 0.57]	430	0.32	[-0.01, 0.69]	336
	0.53	[0.34, 0.74]	457	0.59	[0.38, 0.87]	371

^1^Posterior mean

^2^95% equal-tailed posterior probability interval based on the 2.5^*th *^and 97.5^*th *^percentiles of the posterior density

^3^Effective sample size

**Table 10:** Posterior inference on herd-year-season (co)variances for gestation length (GL) and birth weight (BW) using the bivariate Student's-*t *(BSt) and normal (BN) models.

	BS*t*			BN		
		
Parameters	PM^1^	95% PPI^2^	ESS^3^	PM	95% PPI	ESS
	4.00	[3.02, 5.10]	2,122	4.21	[3.00, 5.60]	1,661
	2.43	[2.04, 2.85]	3,282	2.56	[2.14, 3.00]	3,403

^1^Posterior mean

^2^95% equal-tailed posterior probability interval based on the 2.5^*th *^and 97.5^*th *^percentiles of the posterior density

^3^Effective sample size

**Table 11:** Posterior inference on marginal residual (co)variances for gestation length (GL) and birth weight (BW) using the bivariate Student's-*t *(BS*t*) and normal (BN) models.

	BS*t*			BN		
		
Parameters	PM^1^	95% PPI^2^	ESS^3^	PM	95% PPI	ESS
	51.86	[48.43, 55.84]	2,376	48.90	[47.22, 50.64]	9,039
	3.77	[3.01, 4.56]	8,213	3.20	[2.63, 3.78]	11,671
	13.37	[12.47, 14.41]	2,367	11.14	[10.76, 11.53]	15,789

^1^Posterior mean

^2^95% equal-tailed posterior probability interval based on the 2.5^*th *^and 97.5^*th *^percentiles of the posterior density

^3^Effective sample size

**Figure 1:**
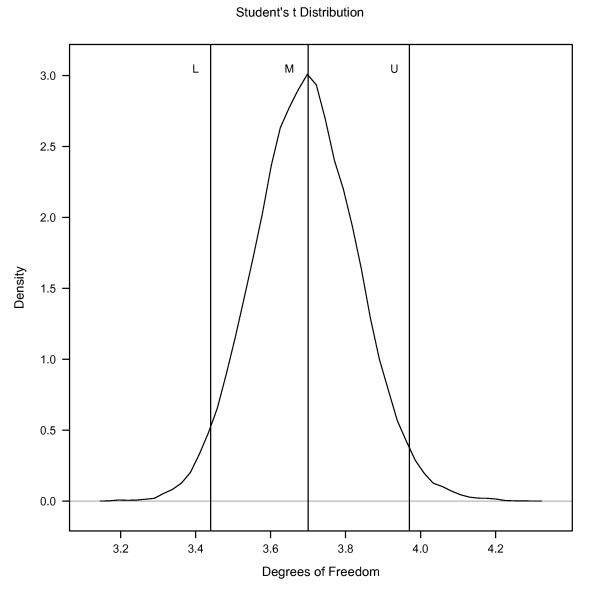
**Posterior densities of degrees of freedom obtained from bivariate Student's-*t *(BS*t*) model fitted to gestation length (GL) and birthweight (BW)**. M represents posterior mean, L represents the 2.5^*th *^percentiles of the posterior density, U represent 97.5^*th *^percentiles of the posterior density.

The posterior distribution of *ν *from the BS*t *model, and its posterior mean (M) and 95% PPI corresponding to the 2.5 (L) and 97.5 (U) percentiles of the posterior distribution are in Figure 1. The posterior mean of *ν *for the BS*t *model was 3.70, with 95% PPI of (3.44, 3.97). This density, characterized by small values of *ν *for BS*t *model confirms that the assumption of normally distributed residuals is not adequate for the analysis of Piemontese GL and BW data.

Posterior inferences on sire-MGS and HYS (co)variances for GL and BW are summarized in Tables 9 and 10, using posterior means and 95% PPIs from BS*t *and BN models. Posterior distributions of (co)variances were nearly symmetric in BS*t *and BN models. Posterior means of sire-MGS (co)variances were similar across models, and 95% PPI widely overlapped. Posterior means of sire-MGS (co)variances from BN model, however, were lower than that from BSt model for GL, and were larger than that from BS*t *model for BW. Covariances from BN model, including sire or MGS effect for BW with sire or MGS effect for GL were higher than those from BS*t *and BS models. Posterior means of HYS variances from BS*t *and BN models were similar and ranged from 4 to 4.25 for GL, and 2.43 to 2.56 for BW from the two models. Posterior inference for the marginal residual (co)variances based on BS*t *and BN models are presented in Table 11. The marginal residual variance for GL, and covariance between GL and BW from BS*t *model seemed to agree with those from the BN model; however, the posterior mean of marginal residual variance for BW from the BS*t *model was significantly higher than that of the BN model.

**Figure 2:**
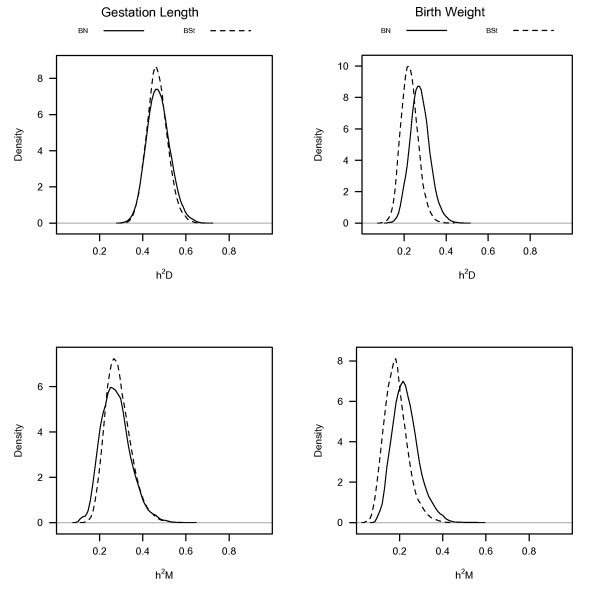
**Posterior densities of direct (D) and maternal (M) heritabilities of gestation length (GL) and birth weight (BW) obtained from bivariate Student's-*t *(BS*t*) or normal (BN) models**. h^2^D and h^2^M represent direct and maternal heritabilities.

**Figure 3:**
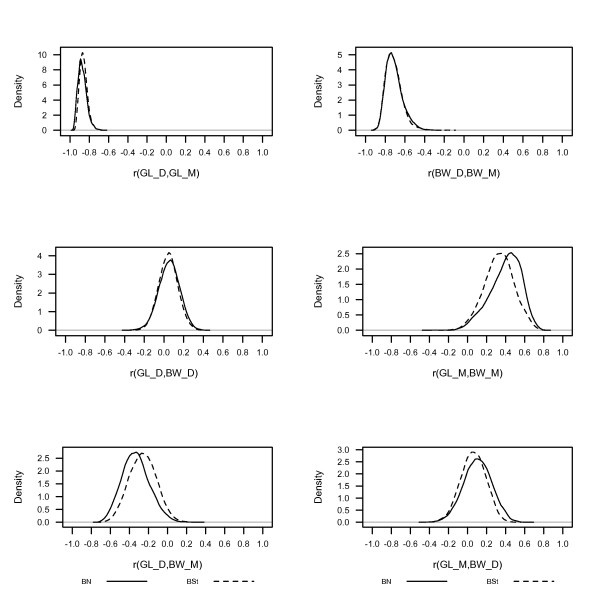
**Posterior densities of genetic correlations between direct (D) and maternal (M) effects for gestation length (GL) and birth weight (BW) obtained from bivariate Student's-*t *(BS*t*) or normal (BN) models**.

Posterior densities of direct and maternal heritabilities, and genetic correlations from BS*t *and BN models for GL and BW are shown in Figures 2 and 3. Posterior means of direct (0.47) and maternal (0.29) heritabilities from BS*t *and BN models were similar for GL. However, posterior means of direct (0.28) and maternal (0.23) heritabilities from BN models were higher than those (0.23 and 0.18) from the heavy-tailed model for BW (Figure 2). In contrast to our findings, Cardoso et al. [7] and Chang et al. [8] have found no real difference in posterior means for heritabilities whether using Student's-*t*, Slash or normal models. Posterior means of direct heritabilities from BS*t *and BN models for GL and BW traits were lower; however, those of maternal heritabilities were higher than the values reported by Ibi et al. [26] and Crews [27]. Posterior means (-0.87, -0.86) of genetic correlations between D and M effects of GL, and those (-0.73, -0.71) of BW from BS*t *and BN models in Figure 3 were significantly negative and very similar with overlapping posterior densities. They were higher than those reported in literature [26,27], and the negative posterior mean of the genetic correlation implies an antagonistic relationship between D and M effects. The posterior densities of genetic correlations between D effects on one trait and M effects on another included zero, indicating non-significant correlations.

**Figure 4:**
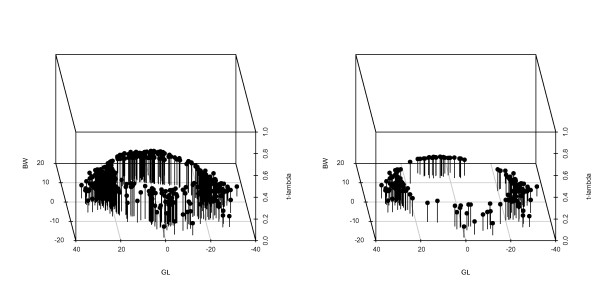
**Distribution of outlier posterior mean values of scale *λ*_*i *_(for each animal) from a Student's-*t *model of residuals plotted against the corresponding estimated residuals for gestation length (GL) and birth weight (BW)**. Distribution of posterior mean values of *λ*_*i *_less than 0.3 on the left. Distribution of posterior mean values of *λ*_*i *_less than 0.2 on the right.

The posterior means of *λ*_*i *_in the BS*t *model can be used to assess the extent to which any particular pair of records presents an outlier for either trait in comparison to a normal error assumption. Low values of *λ*_*i *_(i.e. closer to zero) indicate at least one deviant record among the two traits, whereas values of *λ*_*i *_close to 1 show that the corresponding pair of records match the normal model [17]. The ranges of posterior means of *λ*_*i *_obtained for different animals from the BS*t *models varied between 0.09 and 1.75. The values of *λ*_*i *_are plotted against estimated values of residuals for BW and GL in Figure 4. The distributions of posterior means of *λ*_*i *_less than 0.3 (left figure) or less than 0.2 (right figure) are given in Figure 4. The figure on the right plots posterior mean values of *λ*_*i *_less than 0.2, representing outliers 3 or more standard deviations (SD) from the mean for GL or BW. When the posterior mean values of *λ*_*i *_are close to unity, the estimated values of residuals approach normally distributed residuals, indicating adequate model fit.

In general, random effects contributing to bivariate traits may be correlated positively, negatively or uncorrelated. Accordingly, it is reasonable that effects may vary in their distribution and that residuals for one trait might conceptually be heavy-tailed while others may be light-tailed. Further, it is conceivable that individual animals could exhibit trait specific lambda values. However, in the context of the traits considered in this experiment, it is not unreasonable to imagine that the lambda values could be consistent across the traits because gestation length and birth weight are positively correlated, at phenotypic, genetic and residual levels, and that non-genetic effects that produce residual outliers for one trait such as gestation length might similarly effect birth weight. In fact, single-trait analyses of GL and BW indicate that both traits are heavy-tailed with 2.91 (GL) and 3.66 (BW) for posterior means of *ν*. A more general model that assumes a bivariate distribution for trait-specific *λ *values is more technically demanding than the model used in this paper, but warrants further research.

#### Inference on sire effects

**Figure 5:**
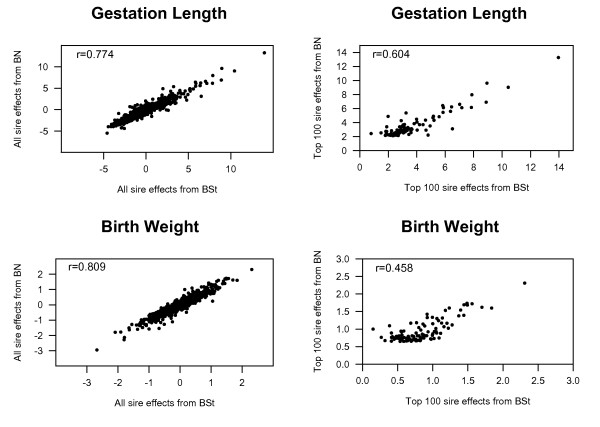
**Scatter plots of posterior means of all and top 100 sire effects for gestation length (GL) and birth weight (BW) in Italian Piemontese cattle, obtained by bivariate Student's-*t *(BS*t*) or normal (BN) models**.

Sire ranking based on posterior means of the sire effects from BS*t *and BN models for GL and BW compared using Kendall rank correlations are in Figure 5. The rank correlation between BN and BS*t *models was 0.77 for GL, and 0.81 for BW, indicating re-ranking of sires among models.

Considering only sires ranked in the top 100 for GL and BW using the BN model, 82% and 75% of them were found to be same for GL and BW in the top 100 animals by BS*t *model. The rank correlations between BN and BS*t *models decreased considerably to about 0.6 for GL and about 0.5 for BW (Figure 5). Cardoso et al. [17] have found similar results in a multibreed genetic evaluation of postweaning gain in Nelore-Hereford cattle and have suggested that a low rank correlation among the top sires may have greater implications for genetic evaluations and selection decisions than the correlation results involving all sires. Figure 5 shows that posterior means of sire effects from BN model shrank to a greater extent under the BS*t *model. Substantial re-ranking of sires was observed due to the greater shrinkage of the posterior mean of sire effects in BS*t *model, and this re-ranking was more pronounced in BW than in GL. Stranden and Gianola [28] have pointed out that animals that are phenotypic outliers will exhibit more extreme predictions of genetic merit under the BN model compared to the heavy-tailed models that mute the effects of the large residuals.

## Conclusions

Bayesian techniques are capable of fitting models where residuals have a heavy-tailed distribution with unknown degrees of freedom. Model comparisons, using PLL, in the simulation study typically favoured the BS*t *models over the BN-∞ model when the true models were heavy-tailed. Further, there was no difference in PLL between BS*t *and BN-∞ models and there were no disadvantages of fitting a BS*t *model when the true model was normal.

Bivariate residual distributions can be assumed normal, or Student's-*t *in the analysis of field data. Predictive log-likelihood values used as model choice criteria in the bivariate analysis of GL and BW data indicated that the BS*t *model with low degrees of freedom fitted better than the BN model. Posterior means of direct and maternal heritabilities from the BN model were similar or higher than those from the BS*t *model. Appreciable differences were observed in sire ranking overall and specifically in the top 100 sires based on rank correlations between BS*t *and BN model sire effects. These results indicate that genetic evaluation and selection strategies will be sensitive to the assumed model. Animals whose *λ*_*i *_values were close to zero in BS*t *model were identified as having one or more outlying records. An interesting extension for future studies would be that of allowing different scale parameter specification for each trait in the BS*t *model.

## Competing interests

The authors declare that they have no competing interests.

## Authors' contributions

KK carried out the simulation and data analysis and drafted the manuscript. DJG and RLF provided support for statistical analysis in the study and helped to draft the manuscript. BM participated in the design of the study and statistical analysis. MAY helped to design and coordinate the study. All authors read and approved the final manuscript.
